# Commercial Value and Challenges of Drop-Based Microfluidic Screening Platforms–An Opinion

**DOI:** 10.3390/mi8060193

**Published:** 2017-06-20

**Authors:** Christian Holtze, Sebastian A. Weisse, Marcel Vranceanu

**Affiliations:** BASF SE, Carl-Bosch-Str. 38, 67056 Ludwigshafen am Rhein, Germany; sebastian-alexander.weisse@basf.com (S.A.W.); marcel.vranceanu@basf.com (M.V.)

**Keywords:** drop-based, microfluidics, screening, commercial value, drop, droplet, high throughput

## Abstract

Developments in High Throughput Screening aim at maximizing the number of samples per time and reducing the cost per sample, e.g., by applying very small sample volumes. The ultimate technological step in miniaturization is moving from microtiter plate wells to droplets, and from batch-wise characterization to the continuous preparation and analysis of samples. A range of drop-based microfluidic screening platforms has emerged that benefit from drop-formation rates of thousands per second, perfect drop size uniformity, plug-flow and compartmentalization, and the possibility of continuously analyzing a train of drops. However, after many years of intensive research, only few commercial applications have been developed and substantial development in the field is still required to make them reliable and broadly applicable. Can academic research achieve this, given that most of the fundamental concepts have been described already, making it hard to publish a big story? Can start-up companies raise enough money to overcome the technical issues of drop-based screening platforms? This contribution addresses the question, focusing on how the different stakeholders in the field should interact so that disillusionment will not put a premature end to the development of drop-based screening technologies.

## 1. History and Introduction to Drop-Based Microfluidic Screening Platforms

Microfluidics is defined as the science and technology associated with the flow of fluids in sub-millimetre channels [1]. The field experienced a first hype in the 1980s, when technologies had been developed that allowed for tailored and reproducible microstructuring. Since this era, only one major application has become commercially successful: inkjet printing. A second hype in the field was initiated in the early 2000s after the invention of soft-lithography—a rapid prototyping technology for the fabrication of microstructures [2,3]. At that time, researchers and industry discovered that flowing immiscible fluids in such structures allows the production of emulsions with an extremely uniform droplet size [4,5,6,7]). Many start-up companies tried to use this capability to grow their business. However, technological issues and the lack of addressable and attractive business needs led to disillusionment in the field. Moreover, by 2010, most of the academically attractive research appeared to have been done in the fields of drop generation and manipulation. Therefore, researchers shifted the focus of their contributions from fundamental research on drop formation and manipulation to the results of applying drop-based screening. Drops were not considered an academic value per se, but they became a tool to generate understanding in the respective field of application. Moreover, most of the conceivable applications of drop-based screenings had been mentioned in publications—an additional sign of an academically mature field.

This contribution will address the question of whether certain applications based on this technology can be developed to be commercially viable or whether the coincidence of commercial disillusionment and the fact that most of the academically low-hanging fruit have already been collected put a premature end to the development.

The most important realization in the community of the mid 2000s was that microfluidics per se is not a value; for commercial success, a microfluidic technology must meet a customer’s need. The field of drop-based microfluidics addresses needs and offers specific value propositions in two main areas: The first of these aims at producing specialty materials. It is based on the capability of making precisely controlled drops and capsules in laminar flow. The key challenge is to realize a robust and massively parallelized process to yield the required amounts of materials. These technologies have been reviewed in [1] and will not be addressed in this contribution. The second field aims at generating valuable results by screening. It is based on the concept that each droplet is an independent vessel, which promises ultra-high throughput at very fast rates, small reagent volumes, high sensitivity, and good control over processing conditions. The key challenges are to realize a robust integrated screening system and establish correlations of the results to conventional screening methods. Prospective applications include the screening of living cells, screening in the fields of diagnostics and sensing, and the characterization of physico-chemical parameters. We systematically assess the fields in which drop-based microfluidic screening can add value. We differentiate three subsets with specific sets of technological challenges and commercial opportunities: screening drop-drop-interactions, screening chemistry within drops, and screening biological systems within drops. For this contribution, we summarize the unique selling points of drop-based microfluidic screening and then present specific examples from these subsets to illustrate the technological benefits and challenges, the key business models we see in the field, and the roles of the respective stakeholders in developing a technology to a commercially viable product. Note that we do not intend to give a comprehensive review of the business and technology landscape, but to share our opinion of what it would take to see more successful drop-based microfluidics in business.

## 2. Technological Unique Selling Points, Advantages and Drawbacks of Drop-Based Microfluidic Screening Platforms

Microfluidic platforms offer advantages over classical methods of screening, when either a relatively small amount of sample is available or the parameter space is extremely big. In order to provide analytical data at sufficient rates, optical detection is most widely used with microfluidic chips made of transparent materials (polydimethylsiloxane (PDMS), glass, polymers like poly methyl methacrylate (PMMA), cyclic olefin copolymer (COC), polycarbonate (PC)). Other sensitive, low-volume analytical methods like nanospray mass spectrometry [8,9], infrared (IR)- and Raman spectroscopy, and even X-ray scattering [10] can be coupled to microfluidic screening platforms in-line. This makes microfluidic screening versatile and broadly applicable [11].

Screening chemical or biological processes or compositions in drops rather than in single-phase microfluidic flow offers many advantages, including no wall-contact and therefore less fouling, perfect plug flow, and no cross-contamination [12]. This makes the microfluidic screening of individual compositions and at individual processing parameters possible. In this way, drops complement classical microfluidics by adding all of the features of vessels and microtiter wells in conventional screening. At the same time, they facilitate a segmented, continuous workflow. Drops can be made at rates of up to 10 kHz and with volumes down to the pico- and even femto-liter regime. Therefore, they offer unprecedented opportunities for screening objects at a low copy number, for expensive materials or materials that are only available in small volumes, and for ultrahigh-throughput. Moreover, microfluidic drop production is unique in that it yields drops of a uniform size. This is necessary for applications that require control over the concentrations and the downstream screening conditions. Finally, a large toolbox for the handling and manipulation of drops in microfluidic processes is available ([13,14]), including mixing chemicals in drops, injecting chemicals into existing drops, realizing the residence time, taking drops off-chip and reinjecting them, sorting drops based on their individual fluorescence intensity, and a wide range of methods of on-chip analysis.

The main drawback of these technologies includes issues related to the robust formation of drops and to interfacial adsorption inside drops: compared to microtiter wells, the surface to volume ratio in such drops is huge, and the interfacial effects can dominate the effects of interest. The leaching of components from the drops and drop destabilization can compromise the test as a whole. A particular challenge in combinatorial screening is the difficulty of generating and handling drops from different sources at very high throughput and low cost: typically, a set of drops is made from a single sample, which may be a library itself. However, it is difficult to generate a library of drops that is composed of hundreds of different sets of drops each containing a different reagent, e.g., for combinatorial drug screening. Another challenge is the fact that the quantities of analyte are very small and consequently require very sensitive analytical equipment. Finally, the drops themselves complicate analysis. In optical detection, lensing effects and scattering at the drop interface need to be taken into account; fluorescence intensity will be switched on and off, depending on whether a drop or continuous phase flows by. The presence of a second, immiscible phase—the continuous phase—will interfere with certain analytical methods. Therefore, it must be separated from the drop-phase of interest before performing the analysis—an operation that has been performed successfully in academic research ([15,16]). This is the case for the fractionated collection of samples to be analyzed by, e.g., chromatography and mass spectrometry.

## 3. Specific Commercial Application Examples

Commercial developments will have to take into account the above inherent technical advantages and drawbacks and focus on one particular unique selling point. The following examples represent the three application subsets described above, in which the effort and risk of development is accompanied by a promising market. We have selected the examples for each of these subsets that we deem to be commercially most relevant.

### 3.1. Example I: Surfactant Screening

Surfactant screening in microfluidics can be carried out along the lines of the papers published by Bibette [17], where droplets are generated in a flow focusing device and afterwards brought into contact by flowing them into a widening channel. Depending on the stabilization that a surfactant system provides, coalescence occurs, either when drops collide, when they are separated from touching neighboring drops, or when drops are subject to cascaded coalescence in closely packed drop arrangements.

The technique offers several advantages over standard coalescence bottle tests uniting two differently dyed drop populations. Firstly, the approach is marker-free—the system under investigation is not chemically altered in any way. Secondly, the method provides insight on a single droplet level, thereby expanding the integral information provided by standard methods. A fact that is especially interesting from an industrial point of view is the possibility to screen many different concentrations and ratios of multiple stabilizing agents in a timely and cheap manner by ramping up and down the relative volumetric flow rates of the respective stabilizer-containing inlet fluids. Microfluidic compositional screening is similar to titration experiments. Therefore, it is superior to the high throughput screening of stabilizer systems in individual vessels or microtiter wells, in which screening large parameter spaces requires many separate experiments, making it tedious and expensive.

Many industries, including cosmetics, nutrition, and crop protection, formulate and sell emulsions and all of them require drop stability. Even though a stability screening apparatus based on microfluidic drop manipulation would promise unprecedented speed and quality in surfactant screening, no commercial system is available. The closest development is Dolomite’s drop merger chip [18], but it is designed to fuse drops of a different composition rather than test stability. The main reason for not having commercialized this promising technology is probably the limited number of potential customers, i.e., development units in the formulating industries. Therefore, at best, such technologies are developed within the industries that develop and sell emulsion-based formulations and are then kept secret to secure their competitive edge. However, product development is also a conservative business and skeptical to new technology: experimental results obtained with microfluidically generated drops that are generally one order of magnitude larger than emulsion drops produced in conventional rotor-stator devices may not directly correlate with the real-world system. Moreover, in practice, specific interactions of the droplet interfaces and the surfactants to be tested with the microchannel walls can induce coalescence that would not occur in bulk. Before a reliable solution to this issue is found, this will limit the applicability and versatility of the method. 

In summary, such screening systems will be used primarily in industrial R&D. The key challenges are related to controlling the drop-drop interactions, while preventing drop-wall interactions altogether. The analytical methods will mostly rely on optical microscopy.

### 3.2. Example II: Segmented Flow Precipitation

Another interesting field in which drop-based microfluidic screening can be applied is parameter screening for precipitation reactions. The drops provide individual segments [19] and realize a perfect plug flow. Backmixing and cross-contamination are not an issue [20]. Each individual droplet acts as a separate reaction compartment in which many parameters can be varied. These parameters include the temperature, concentration of chemicals, and chemical composition. The associated physicochemical parameters, e.g., nucleation rates, can be quantified. Such methods have been successfully applied to optimize the composition and processing parameters for the development of quantum dots [21]. Moreover, as large numbers of drops and therefore large numbers of separate experiments can be performed in a short time, the method is suited to the generation of statistically relevant data. Screening large parameter spaces quickly and with very small amounts of material is particularly appealing for the characterization of the precipitation and crystallization processes of proteins. This has been demonstrated by nucleating and growing single protein crystals inside drops [22].

In some cases, in which solid formation gives rise to fouling issues and the clogging of microchannels, drop-based microfluidics can even enable microfluidic screening: precipitation takes place inside the drops and—provided the system is properly set up—the solids that form never even touch the microchannel walls. Consequently, the screen runs robustly in a wide parameter-space.

Additionally, for optimizing precipitation conditions, to the best of the authors’ knowledge, no screening device is commercially available. The reasons for this are again the limited number of potential customers and skepticism toward new technology in the R&D divisions of companies. Most of the industries that sell precipitated and crystallized materials develop their processes based on conventional batch-experiments and on their experience. Products are often post-treated by milling and sieving to obtain the desired particle size for the application, rather than modifying the precipitation process itself. Therefore, a fast and reliable screening of the precipitation process would have the potential of significantly improving the product quality and reducing the workup cost, but to be successful and accepted, its results must be translated into an improved process on an industrial scale. However, experience shows that the products obtained in large vessels are often different from the products that would be expected based on lab tests or even microfluidic tests, or from thermodynamic or theoretical considerations. In large-scale precipitation traces of impurities or seed crystals, this can have a dominant effect. Therefore, product developers are concerned that the dominant species is not clear from or not even found in drop-based experiments. As a prerequisite of successful screening, it will be necessary to conduct fundamental research on the experimental methodology and on the correlation between microfluidic and large-scale precipitation. With such expertise, an expert service, rather than selling a benchtop device, could add substantial value, for example, in the pharmaceutical industry. Their actives often need to be precipitated with very good control as polymorphism and co-crystals are relevant in registration and patenting.

In summary, precipitation and crystallization screening systems will also be used primarily in R&D for chemical/material synthesis. The key challenges are related to preventing contact between the chemicals inside the drop and the microchannel walls and to implementing meaningful analytics. For the general screening of chemical reactions, a range of analytical methods will be required to yield the chemical information of interest, and desirable methods include mass-spectrometry and nuclear magnetic resonance (NMR) spectroscopy, IR, Raman, UV/VIS-spectroscopy, gas and liquid chromatography, and scattering methods [11].

### 3.3. Example III: Biological Screenings

Drop-based microfluidic screening quickly gained interested in biology and in the life sciences [23]. The virtues of the technology and the needs in the field match nicely as such screens require very small amounts of mostly expensive samples and chemicals, e.g., buffers or substrates. Given that these methods are suited for ultrahigh-throughput screening, large parameter spaces become accessible. Moreover, the typical materials used in the published literature are biocompatible. Commercial applications mostly target biomedical applications like cancer screening, microtoxicology, diagnostics, and sensing that benefit from the technological features of drop-based microfluidics.

The field of diagnostics and sensing has been covered widely in the literature [24]. Here, the robustness of microfluidic platforms and low cost of devices are the crucial features. A prominent example, in which drop-based microfluidic screening techniques can offer a unique advantage, is digital polymerase chain reaction (PCR), which features an increased sensitivity through the compartmentalization of individual DNA molecules. Raindance Technologies have developed a benchtop digital PCR-system based on microfluidically produced drops. Alternative pieces of equipment that are based on the same concept and aim at genome sequencing are the Chromium product line by 10× Genomics and the Bio-Rad QX200. We are not aware of any other commercial application in which drop-based systems have been developed to an involved, easy-to-use benchtop system. We believe that this is because technological alternatives that do not rely on drop-based microfluidics appear to be easier to develop and more robust in operation.

Other fields of interest are ultrahigh-throughput cell screenings and toxicological screenings [25]. Drop-based microfluidics offer the unique possibility to work on a single cell level and they do not generate integral ensemble data. They allow one to investigate the interaction of chemicals with individual cells or with the metabolism/biochemistry on the single cell level, given that a segmented flow offers the opportunity to create single compartments with individual chemical compositions and to investigate the response to process parameters like temperature, light, and residence time in a straight forward fashion. While this is very attractive academically, a main drawback for commercialization is that it is not clear how the results of such screenings correlate with the results observed in the real-world. Their cells usually appear in ensembles, which can totally change their behavior with respect to that of a single cell.

Protein analytics can benefit from techniques that separate and fractionate proteins from solution into individual drops. This has been demonstrated by using a combination of isoelectric focusing and the slipchip technology, which highlights that the mechanism of drop formation in microfluidic setups does not always have to rely on the flow of immiscible fluids at microchannel junctions [26].

Many involved micro-total analytical systems (µ-TAS) are ambitious long-term research projects that are still in the stage of academic research. One example that requires much of the available know-how on handling drops is related to the directed in vitro evolution of enzymes for the biosynthesis of chemicals [27,28]. In this research, the drop acts as a compartment, in which a cell and all the chemicals it produces through its metabolism are contained. The objective is to screen for cell strains and/or individual clones which are producing a desirable chemical at a high yield and selectivity, and to select these strains or clones. Cloning and proliferation will provide cells that make biotechnological processes like fermentation more profitable.

A major challenge for both in vivo and in vitro biological screening is the choice of the appropriate chemicals. Fluorocarbon oils are widely used, given that they are biocompatible and that they act as a good barrier against cross-contamination, even for lipophilic components. Moreover, the choice of the surfactant will be crucial to the experiment [12]: it must prevent the adsorption of cells or biomolecules, which would lose their tertiary structure, and at the same time, it must ensure stability against the coalescence of drops if they hit each other on the chip or if they are incubated off-chip. Such stabilizers are an example of tailored chemicals that enable the development of drop-based screenings. They are commercially relevant in that they are IP-protected and serve as important differentiators in the market. At the same time, their limited supply and often high sales price slows down novel academic developments.

Working with drops in microfluidics requires engineering and physics skills. This makes it hard for interested biologists and chemists to perform their experiments in this way, if they do not want to spend the time learning a new technology. An interesting development in this sense is the Dolomite Mitos Dropix system [29]. It provides a range of drop generation and handling operations that are relevant to biological experiments, e.g. immunoassays in drops in a benchtop system that are easy to use for non-specialists. Operating instructions and suitable chemicals are also available from the supplier. The drawback, as always, is that the cost associated with this system will limit its widespread application, particularly in academia.

In conclusion, the largest and most visible market of biological screening systems will be point-of-care tests. Key challenges for these fields are the ease of handling and robustness. The most important analytical methods will rely on fluorescence detection.

## 4. Commercial Considerations and Possible Business Models

In developing a business with drop-based microfluidic screenings, various stakeholders must be considered (see Table 1). First, the consumer must have a need and be willing to pay a price that makes the business profitable. The price that they are willing to pay will be strongly influenced by non-microfluidic or non-drop-based screening alternatives. Second, the vendor/instrument manufacturer of the screening will have to ensure the supply, robustness, and quality of the product, and will need to worry about liability and the appropriate access to the market, ideally through an established marketing network or a vendor. Third, for robust production, in many cases, chip manufacturers will supply the devices that are eventually sold to the customer. As the development of robust microfluidic technology is closely linked to chip manufacturing, many chip suppliers have established development services for their customers. The success of such a collaboration will depend on short response times during development and reliable supply, once the product is commercialized. Fourth, the role of academia is to generate novel screening concepts and provide a toolbox of robust microfluidic drop handling and manipulation protocols.

Depending on the application, the consumer can be an individual user like a patient, an analytical lab in a big hospital, or an R&D facility in a large corporation. This will determine whether the main value is generated by selling disposable chips; by providing a package of the analytical setup, chips, and assay materials for unique diagnostic analyses; or whether the value of a proprietary microfluidic screen consists of saving time and money by speeding up product development. In the latter case, one business-model can be employed for providing a service.

It is the interplay between market needs and technological feasibility that determines the type of business of a particular microfluidic test. For example, a diagnostic market need is defined by alternative analytical options, customer preference, and the market size (number of tests), while the technological feasibility can be determined by the shelf life of a key ingredient, expensive infrastructure for read-out, or the required qualification of the operator/evaluator. Consequently, only very few tests will qualify for end consumer use as such tests must be extremely robust and simple to perform. Without even considering the cost of registering such tests with the authorities, their development will be much more expensive than the development of a typical R&D screening method for in-house use.

## 5. Developments in the Field and Recommendations

### 5.1. Market Perspective

The above collection of methods and applications illustrates that from a commercial point of view, the interesting fields of work have to be developed by academia to a point, where background knowledge and the methods have been demonstrated with model systems. The crucial part required to bring these techniques to use in a commercial environment is applicability. This means that known concepts have to be transferred to real-life systems and into robust and reliable methods and standard operating procedures. This will enable the stakeholders involved in the development of chips and instruments, as well as R&D-users, to benefit from the advantages of drop-based microfluidics, without the need of time consuming and expensive method development. The following section classifies different stakeholders in the field and discusses their needs and interactions based on the developments and trends in the community.

### 5.2. The Stakeholders

#### 5.2.1. Academia

In the first decade of this millennium, most of the tools used to produce, handle, and manipulate drops in microchannels were developed. Such challenges were primarily tackled by engineers, and physicists developed concepts for understanding the fundamental fluid dynamic processes. Over the past couple of years, the community has realized that most of the necessary and relatively straightforward research has already been published. Therefore, it changed its focus and uses such concepts as tools, e.g., for biological studies. Engineering-type microfluidics-related questions are increasingly perceived as a hassle rather than as an interesting academic challenge. In parallel, the engineers and physicists have shifted to need-driven research and tackle involved challenges like developing the simple and reliable rapid prototyping of microfluidic chips in other materials than PDMS, alternative drop-maker geometries, or drop sizes of less than 10 µm [30], which are at best, incremental improvements and by no means conceptual break-throughs. Consequently, by now, the greatest fraction of publications focus on the results of the drop-based screening of biological phenomena, rather than the method. This paradigm-shift is indicative of a mature field. It is a healthy development, as academic research needs to inspire an industry with ideas and concepts for new applications and spin out start-ups with novel and unique value propositions.

#### 5.2.2. Chip Manufacturer Industry

Along with the enthusiasm for microfluidics in academia, a number of small start-up companies were started, while the larger manufacturing industries in glass, injection molding, and hot embossing observed the market, but did not immediately participate. As the small entities were generally started based on one core technology and the need to focus, they had a very strong focus on their proper technology—which did not necessarily benefit the customer/vendor of an end-consumer product. The market has seen four trends since these early days: consolidation, specialization, diversification, and integration. As a consequence of consolidation, some players have vanished, whilst others have been acquired or merged (Little Things Factory, http://ltf-gmbh.com/). Specialization accompanied by considerable investment has created foundries which focus on tailored chip production with very good reproducibility (Industrielle Masken und Teilungen, https://www.imtag.ch/en/). Diversification is the most logical answer to customers’ needs of an organically growing company: In addition to tailored, customer-specific chip manufacturing, many companies offer standard geometries off-the-shelf (Micronit, http://www.micronit.com/; Chip Shop, http://www.microfluidic-chipshop.com/). In some cases, classical glass-manufacturers even offer chips made of polymers, even though up to date companies generally either focus on glass (Micronit, IMT, LTF, Dolomite) or on polymers (Chip Shop, Sony DADC)—a one-supplies-all supplier does not yet exist to the best of our knowledge. An interesting case of diversification is forward-integration into the domain that would typically be covered by the customers of chip foundries: chip manufacturing is complemented by the engineering of benchtop and handheld apparatus and licensing agreements for IP relating to drop formation. (Dolomite, http://www.dolomite-microfluidics.com; Syrris, http://syrris.com). Finally, integration is a fairly common trend, where big companies aiming at making business with microfluidic chips acquire attractive chip supplier companies. Böhringer-Ingelheim acquired microParts [31], Bayer acquired Ehrfeld [32], and Stratec acquired the relatively young microfluidics branch of Sony DADC [33]. These examples suggest that microfluidic chip manufacturing is by no means a mature field and that big players see it as a key distinguishing competence in their product development.

We expect these trends to continue over the next decade. Only incremental change will be seen for the glass-microfluidic supplier market, which in 2017, already appears to be consolidated and global. Much more change is to be expected for the polymeric chip providers, who have mostly settled on polycarbonate and COC (cyclic olefin copolymer) that can be processed with standard injection molding equipment. Therefore, established players for precision injection molding and hot-embossing having both equipment and expertise at hand try to enter the microfluidics market. This makes a comprehensive overview of polymeric chip providers almost impossible. When it comes to the surface-functionalization of polymeric chips as an additional, distinguishing criterion, it still seems to be very hard to identify the supplier of choice. In this case, however, it is because the community does not seem to have identified and settled on one robust and reliable technology. Consequently, many developments are tailored to one customer and become quite time-consuming and expensive, hindering the development and commercialization of disposable microfluidic screening chips.

#### 5.2.3. Instrument Manufacturer Industry (Original End Manufacturer OEM)

The vendors of microfluidic screening platforms, who do not practice the in-house development and fabrication of microfluidic chips, rely on a relationship of mutual trust with their chip supplier. If a supplier oversells his capabilities, reproducibility, or the number of parts, he puts a major threat to the vendor, who generally takes the risk of product development. Vice versa, vendors can seriously affect suppliers, who invest in large production capabilities that might never be used due to wrong market forecasts. This may have been an additional motivation for the above-mentioned acquisitions of supplier companies [31,32,33].

#### 5.2.4. Distributors

In many cases—predominantly in point-of-care testing—an intermediate stakeholder will take care of the distribution of the instrument. Distributors are necessary if the instrument provider does not have a marketing and sales force to directly interact with the people that operate the instrument. For example, distributors sell or rent and maintain the instruments for pharmacists and doctors that offer point-of-care testing as a service to their patients or end-consumers that monitor health parameters with a handheld device at home. 

#### 5.2.5. Service Companies

A range of companies offer services and consulting to researchers in academia, start-ups, and industrial R&D. This ranges from turnkey solutions for setting up one’s own soft-lithography lab (Blackholelabs, http://www.blackholelab-soft-lithography.com/) to design and engineering support for benchtop or handheld microfluidic systems (Syrris). User-friendly equipment is available for researchers interested in performing specific drop-based operations and even certain workflows comprising several operations, without having to be educated in chip manufacturing and handling nor drop formation and analysis (Dropix by Dolomite [29]). Such services are complemented by classical technology consulting with a strong focus on microfluidics (Enabling MNT, http://www.enablingmnt.co.uk/).

#### 5.2.6. Customer

The customer base for drop-based microfluidics can be divided into two classes: end consumers, e.g., patients that need a point-of-care test, and the users of drop-based microfluidics for R&D: 

Customers that purchase point-of-care tests and patients in hospitals will at best benefit from the improved speed, quality, and price of novel diagnostic tests. The responsible individuals in hospitals, analysis labs, and health-care systems or distributors will take the decision of whether to introduce a drop-based microfluidic test or not, based on these criteria. Therefore, commercial success in many cases will not depend on end-consumer decisions but on the decisions of these intermediate stakeholders that will be driven mostly by cost and regulatory requirements. For this reason, we advise to focus on their needs of building a business model for novel screening, speed, and convenience which determine that the user experience will matter most for products that are directly operated by end-consumers, e.g., in lifestyle point-of-care testing.

Customers that work as developers in industrial product development, however, will be affected most by powerful development tools. In order to succeed in this market segment, it will not only be important to convince them that the novel tool generates results that correlate with their application tests, it will also be important to show best-practice examples, in which the new screening technology outperforms the conventional ones. However, given that many of these examples are considered proprietary, and that—if they are successful—they are most likely kept as an industrial secret, we expect that it will take a while until convincing arguments can be given for why product development should apply novel technology.

### 5.3. IP and Standardization

Instrument Manufacturers (Vendors) developing microfluidic technologies have been facing a very confusing intellectual property (IP)-situation, where freedom to operate was and is hard to predict. This is because some of the fundamental inventions in the field of drop-based microfluidics have not come off-patent yet, as they were made after the beginning of this millennium [34]. This is because of the vast number of patents that have been filed in the past years and the fact that a final decision by the patent offices is still pending for many of them. It is aggravated by some stakeholders holding large portfolios of IP (Caliper Life Sciences [35]), who are willing to sue even small start-up companies infringing with their IP. We expect the IP-situation to be much better in the mid-2020s for new developments and for generics, which will boost commercial activity.

The issue of standardization has been raised many times in various contexts. Vendors will object to standards for their disposables, because of customer retention. However, the registration of new products with the authorities will be crucial. Only by vendors joining forces and by establishing standards, mandatory statistics, and best practice can approval by the administration be demonstrated. The Japanese microfluidic vendor community seems to have been the first one to collect arguments for registration in a joint effort.

## 6. Opinion

Microfluidic screening technologies based on single-phase flow are clearly emerging. The value in point-of-care diagnostics and related fields is generally accepted; they are being developed by many small and large companies. Developments are supported by a range of suppliers for suitable equipment regarding the infrastructure and functional microfluidic devices, and some even specialize in engineering support. While drop-based screening technologies can benefit from these developments, they face one additional technological challenge: proper dealing with the liquid-liquid interface between the drop and the continuous phase, which in combination with ubiquitous microchannel walls, makes this an essentially three-phase system. Exquisite control of the interfaces will require additional competence in interfacial chemistry and functionalization in a product development team that already has to deal with microfluidic issues on top of all the application-related challenges. This drives cost can result in longer development times and adds risk to the development. Moreover, once a drop-based screening platform is developed, in many cases, it will be limited in its versatility or robustness, if under certain conditions, components in the test-system adsorb to the interfaces and interfere with the test. Consequently, this restricts the addressable market size and hence the revenue for a specific development. 

Undoubtedly, drop-based screening platforms promise many benefits and will be an important pillar of future products. However, they are still in their early days as they require interfacial control of both the drop and the channel wall interface. Given that drop handling is associated with a higher risk in product development and restrictions in the addressable market, we recommend their development only if they promise a clear competitive edge over single-phase microfluidic screening. We see the main unique value propositions in the three areas exemplified above: (1) screening the interaction between drops, e.g., for stabilizer selection; (2) screening (possibly) solid forming chemical reactions, in which drops prevent direct contact with the microchannel walls and hence improve robustness; and (3) performing biological experiments, in which the key benefits are the reduced consumption of chemicals, compartmentalization of cells and cell components, and ultra-high throughput screening.

With growing experience, best practice examples, and possibly a better supply of enabling chemicals and microfluidic drop-production and handling solutions, we will see many drop-based technologies emerge. However, this will be driven by a need for further improvement and differentiation and will therefore only occur after single-phase microfluidic screening is well-established. Consequently, we expect to see only a few and very specific successful commercial developments of drop-based technologies in the next years. In academia, we expect increasingly application-driven research in a few groups worldwide, that at the same time master the challenges of drop-based microfluidics and the chemical or biological expertise required by the application of interest. A huge opportunity for speeding up this projected evolution would be the development of rapid, reliable, robust, and inexpensive surface-functionalization for drop-production in chips, ideally patent-free and ready-to-use in combination with soft-lithography. This would greatly broaden the user community by giving non-experts access to drop-based developments.

## Figures and Tables

**Table 1:** Overview of the stakeholders and their roles and interactions in bringing microfluidic screening technologies to the market.

Customer/User	Application Example	Business Model	Key Criterion	Vendor Industry	Supplier	Input from Academia
End consumer	Pregnancy test, diabetes test	Sell devices	Robust, mass producible	Pharmaceutical and analytical industry	Automated, certified mass production	Collaboration on specific technological challenges
Hospitals, Industrial R&D, Centers for analysis	Advanced blood testing, cancer diagnosis		Infrastructure (chemicals and devices)			
Governmental authorities, Industrial R&D	Toxicological profiles	Offer service	Complex handling	Analytical service labs, start ups	Partly manual manufacturing	Spin off start-ups, solution to crucial processing steps
Academia, R&D in large industrial corporation	Tool to speed up development of novel enzymes^+^	Develop in house	Specific application, differentiator in market	Academia, R&D in large industrial corporations	Rapid prototyping	Concept, Collaboration on specific aspects
Academia	Understand mechanism of drop coalescence	Academic interest, no business	Nitch market, understanding	Academia		Realize entire workflow, demonstrate feasibility
